# Analysis of Physiological Variations and Genetic Architecture for Photosynthetic Capacity of Japanese Soybean Germplasm

**DOI:** 10.3389/fpls.2022.910527

**Published:** 2022-06-29

**Authors:** Mohammad Jan Shamim, Akito Kaga, Yu Tanaka, Hiroshi Yamatani, Tatsuhiko Shiraiwa

**Affiliations:** ^1^Laboratory of Crop Science, Division of Agronomy and Horticultural Science, Graduate School of Agriculture, Kyoto University, Kyoto, Japan; ^2^Institute of Crop Science, National Agriculture and Food Research Organization, Tsukuba, Japan

**Keywords:** soybean core collection, gas exchange, K-means clustering analysis, GWAS, RNA expression

## Abstract

The culmination of conventional yield improving parameters has widened the margin between food demand and crop yield, leaving the potential yield productivity to be bridged by the manipulation of photosynthetic processes in plants. Efficient strategies to assess photosynthetic capacity in crops need to be developed to identify suitable targets that have the potential to improve photosynthetic efficiencies. Here, we assessed the photosynthetic capacity of the Japanese soybean mini core collection (GmJMC) using a newly developed high-throughput photosynthesis measurement system “MIC-100” to analyze physiological mechanisms and genetic architecture underpinning photosynthesis. K-means clustering of light-saturated photosynthesis (*A_sat_*) classified GmJMC accessions into four distinct clusters with Cluster2 comprised of highly photosynthesizing accessions. Genome-wide association analysis based on the variation of *A_sat_* revealed a significant association with a single nucleotide polymorphism (SNP) on chromosome 17. Among the candidate genes related to photosynthesis in the genomic region, variation in expression of a gene encoding G protein alpha subunit 1 (GPA1) showed a strong correlation (*r* = 0.72, *p* < 0.01) with that of *A_sat_*. Among GmJMC accessions, GmJMC47 was characterized by the highest *A_sat_*, stomatal conductance (*g_s_*), stomatal densit*y* (*S_Density_*), electron transfer rate (*ETR*), and light use efficiency of photosystem II (*Fv’/Fm′*) and the lowest non-photochemical quenching [*NPQ*(*t*)], indicating that GmJMC47 has greater CO_2_ supply and efficient light-harvesting systems. These results provide strong evidence that exploration of plant germplasm is a useful strategy to unlock the potential of resource use efficiencies for photosynthesis.

## Introduction

It is estimated that the current rate of global crop production needs to be doubled to meet the demands of the rising population, changing diet, and requirement for biofuel (Tilman et al., 2011). Although crop seed yield has increased in many parts of the world, its stagnation in an estimated 24–27% of the areas, where maize, rice, wheat, and soybean are grown, underscores the challenge to address the rising demands for agriculture (Ray et al., 2012). Climate change including, rising temperature, drought, flooding, and air pollution further aggravate this challenge (Christensen and Hewitson, 2007). With the decrease in arable land and resources, developing novel strategies to maintain sustainable plant production for the food and energy demand of humanity is essential (Ray et al., 2012; Maurino and Weber, 2013). Conventional breeding and selection of cultivars have been used to improve yield and it is estimated that rice and wheat have acquired ~1% annual gain in yield potential in the last 30 years (Stapper and Fischer, 1990; Khush, 2001). However, recent studies have reported the exhaustion of the traits that led to a remarkable increase in crop seed yield during and after the green revolution (Zhu et al., 2010). An effective way of producing sufficient food, practicing sustainability, and avoiding the exploitation of natural resources is suggested to be the manipulation of photosynthesis (Janssen et al., 2014).

Improving photosynthetic capabilities in plants, however, is a complex process and requires the removal of inefficiencies from the photosynthetic machinery. Photosynthesis is majorly limited by biochemical and diffusional factors and several targets have been proposed for genetic manipulation to increase photosynthetic capacity. For instance, copious quantities of Rubisco that catalyze two competitive processes of CO_2_ fixation and oxygenation are required for better photosynthetic capacity. Changes in the quantity, specificity, or affinity of CO_2_ in Rubisco can boost photosynthesis (Parry et al., 2012). Reduction in transient photoinhibition, increase in ribulose 1,5-bisphosphate regeneration capacity, and increase in specificity of Rubisco for CO_2_ have been considered ideal targets for the genetic manipulation of light-saturated photosynthesis in C3 crops (Lawlor et al., 1999). The overexpression of chloroplast NAD kinase (NADK2) was observed to play an important role in photosynthetic electron transfer thus improving photosynthesis (Takahara et al., 2010). Similarly, a study on transgenic *Arabidopsis thaliana* revealed that increased stomatal density (maximum 372%) increased the photosynthetic rate by 30 percent (Tanaka et al., 2013). The genetic manipulation of *g_s_* in *slac1* mutant (lacking stomatal anion channel protein that controls stomatal closure) revealed that a higher *g_s_* enhances the rate of photosynthesis as well as the ratio of internal to external CO_2_ concentrations (Kusumi et al., 2012). Mounting evidence suggests that the genetic manipulation of rate-limiting processes enhances photosynthesis. The problem, however, is that a large part of plant germplasms remains unexplored. Harnessing natural variations in photosynthesis, therefore, is an ideal route toward removing bottlenecks in photosynthesis.

The exploration of plant germplasms for identifying promising accessions and elucidating the association between genetic architecture and photosynthesis is important to enhance photosynthesis. Kromdijk and Long (2016) stated that there is a large gap between the number of germplasms preserved in gene banks and those used in crop breeding. They argue that delayed photosynthetic improvement is largely due to the plant germplasm that remains unexplored. The exploration of core collections is, therefore, essential for identifying new targets to use in crop improvement (Upadhyaya, 2015). On the other hand, studies have been conducted to assess the possibility of enhancing photosynthesis through genomics and gene modification. Lopez et al. (2019) reported a high genetic correlation (*r* = 0.80) between seed yield and photosynthesis in the US soybean nested association map (NAM) panel. They also found single nucleotide polymorphisms (SNPs) which were associated with photosynthesis. Vieira et al. (2006) identified some quantitative trait loci (QTLs) on chromosomes 10 and 16 that were associated with light-saturated rates of photosynthesis. Simkin et al. (2015) indicated that increasing the levels of sedoheptulose-1,7-bisphophatase, fructose1-,6-bisphosphate aldolase, and the cyanobacterial putative-inorganic carbon transporter b (ictB) improved photosynthetic CO_2_ fixation (12–19% in mature plants), leaf area, biomass, and yield in transgenic tobacco. Xiao et al. (2020) found that heterologous expression of the highly linked genes (*PtoPsbX1*) in their gene expression network significantly improved photosynthesis in *A. thaliana*. These findings collectively suggest that exploring the plant germplasms for natural variations in photosynthetic capacity and studying the genetic architecture underlying photosynthesis is an ideal strategy for improving photosynthetic efficiency in plants.

Soybean is a major commodity crop (Hay et al., 2017) and an indispensable part of the Japanese diet; it is also said to increase longevity among Japanese people (Gabriel et al., 2018). Japanese soybean cultivars, however, are less productive than the US cultivars, as the latter have higher radiation use efficiency (RUE), greater photosynthetic capacity, and dry matter production (Kawasaki et al., 2016). GmJMC (JMC hereinafter) is a small but diverse germplasm set named mini core collection that represents a major part of geographical and agro-morphological trait variations of Japanese soybean landraces (Kaga et al., 2012). It is consisted of 96 accessions selected from 1,603 soybean accessions based on genetic variation. However, JMC has never been evaluated for its genetic variations in leaf photosynthetic capacity. Considering the geographical and agro-morphological characteristics of this collection, we assume that studying leaf photosynthetic capacity would divulge important information and provide suitable targets for resource use efficiencies. This study aimed at assessing the photosynthetic capacity of JMC accessions using a newly developed high-throughput photosynthesis measurement system, MIC-100, by analyzing physiological variations and genetic architecture underlying photosynthetic discrepancies and identifying suitable targets to improve photosynthetic efficiency.

## Materials and Methods

### Plant Materials

All germplasms were obtained from National Agricultural and Food Research Organization (NARO) Genebank (Japan). Two experiments were conducted for this study. The first experiment was aimed at assessing phenotypic variations in *A_sat_*. We selected 90 accessions from the JMC and sowed their seeds on 18 June 2019; among which 16 accessions revealed lodging and poor seed quality. The remaining 74 accessions were cultivated again on 20 June 2020 using a randomized complete design (RCD). Ten plants were sown in each plot.

The second experiment was conducted in 2021 to measure various parameters related to photosynthetic capacity in 10 representative accessions (indicated by white dots inside markers) and investigate the mechanisms underlying it. We selected seven-candidate accessions (JMC13, JMC16, JMC37, JMC43, JMC47, JMC49) from the best photosynthesizing Cluster2; two accessions (JMC56, and JMC112) from Cluster1; one accession (JMC25) from Cluster3; and a Chinese accession Peking (named “PE” as a check for having higher *A_sat_* and *g_s_*). JMC25 and JMC112 are two photosynthetically elite cultivars, named Enrei (“En”) and Fukuyutaka (“Fu”), respectively. These accessions were cultivated in a randomized complete block design (RCBD) with two replicates.

All experiments were conducted in a field at the Laboratory of Crop Science, Graduate School of Agriculture, Kyoto University (35°0.2” N, 135°. 47″ E). The sowing density was 9.52 plants m^−2^. The space between rows and between plants in a row was 0.7 m and 0.15 m, respectively. We applied 3:10:10 gm ^−2^ of N:P_2_O_5_:K_2_O fertilizers during sowing. Three seeds were planted per hill of which only one was retained after unifoliate leaves had fully expanded. Irrigation and pesticide application were done regularly.

### Photosynthetic Phenotyping

We used a high-throughput portable close-chamber infrared gas analyzer, MIC-100 (MASA International Cooperation, Japan), to analyze light-saturated photosynthetic rates (*A_sat_*) in 2019 and 2020. This system requires less than 30 s per sample whereas a standard LI-6800 achieves stability in at least 45 s for the “Fast Survey Measurements.” MIC100 explains around 95% variations in *A_sat_* measured by a standard LI-6800 system (LI-COR, United States) therefore, making it a high-throughput efficient system (LI-COR Biosciences, 2021; Tanaka et al., 2021). Moreover, our observations in the field revealed that MIC-100 can measure *A_sat_* up to sevenfold faster than LI-6800. Measurements were conducted for two consecutive years: three measurements in 2019 (5 August, 13 August, and 18 August) and five measurements in 2020 (06 August, 14 August, 21 August, 27 August, and 31 August). The data were collected from 9:00 AM to 1:30 PM from a central leaflet of a fully expanded recently matured trifoliolate from flower initiation stage (R1) to seed initiation stage (R5). We were able to measure *A_sat_* in 360 and 240 leaves on each measurement day for 2019 and 2020, respectively which collectively sums up to 2,280 samples. Only one plant was sampled at a time so that *A_sat_* is distributed throughout the survey time to compensate for the diurnal privilege of accessions. Light intensity inside the cuvette was 1,200 μmol m^−2^ s^−1^ which is the maximum capacity of this system. Temperature is automatically recorded at the end of each experiment and did not exceed 36°C. Ambient CO_2_ and humidity conditions were used for the experiment, with the initial CO_2_ concentration set at (370–390 ppm) and the final concentration set 20 points lower than the initial concentration.

In 2021, an LI-6800 (LI-COR, United States) was used on 22 July, 30 July, and 11 August for gas exchange and chlorophyll fluorescence measurements. These parameters were measured in four randomly selected plants from each plot from 9:00 AM to 11:00 AM. The temperature inside the chamber cuvette was 33°C. Reference level CO_2_ was set at 400 μmol mol^−1^ using CO_2_ cartridges, with an airflow rate of 500 μmol s^−1^. Light intensity was set to 1,200 μmol m^−2^s^−1^ with 10% blue light and relative humidity was set at 60%. We calculated NPQ(t), as an alias to NPQ, following Tietz et al. (2017).

### K-Means Clustering Analysis

Before performing K-means clustering analysis, we used T-distributed Stochastic Neighbor Embedding (t-SNE) to reduce the dimensionality of the data (*A_sat_* was measured 8 times throughout 2019 and 2020). The data were standardized so that each measurement had a mean of zero and a standard deviation of one. The number of components in t-SNE was set to two and we gave it 1,000 iterations. The output data were used in the K-means clustering algorithm with four centroids.

### Genome-Wide Association Study

The SNP dataset obtained for 192 soybean accessions of mini core collection (Kajiya-Kanegae et al., 2021) was used in this study. The SNPs dataset with minor allele frequencies of more than 5% for the 74 accessions was filtered using PLINK 2.0 (Chang et al., 2015). Association tests between the t-SNE and the SNPs dataset were conducted using a function of “association.test” in gaston package ver. 1.5.7 in R (McKenna et al., 2010). First, five principal components were included as fixed effects in the linear mixed model. A genetic relationship matrix (GRM) and ρ values of the marker-trait associations were calculated using the “GRM” function and Wald’s test, respectively. Manhattan plot and QQ plot were drawn using the “manhattan” and “qqplot.pvalues” functions, respectively. The genome-wide significant threshold was obtained based on a false discovery rate (FDR) at a 5% level using the “p.adjust” function in R (Benjamini and Hochberg, 1995).

### Gene Selection

We selected all the genes in a 250 kbp region from both sides of the significant peak detected in GWAS together with annotations from the reference genome sequence Gmax_275_v2.0 from Phytozome (Phytozome v.12, Supplementary Table S1). About 19 genes that had either photosynthesis-related functions or higher expression in photosynthesis-related plant tissues were selected based on the Phytozome database search. The SNP data and Indels in these genes among the 74 accessions were extracted from Illumina read mapping data (Kajiya-Kanegae et al., 2021) using CLC Genomics Workbench 12 (Qiagen, Germany).

### RNA Expression

On 24 August 2021, we measured the *A_sat_* of the central leaflets of 14 randomly selected JMC accessions and took about 10 mg of a fresh tissue sample for gene expression analysis immediately after the *A_sat_* measurement. Leaf samples were frozen in liquid nitrogen and stored at −60°C. Total RNA was extracted by TRI Reagent (Molecular Research Center, United States). First-strand cDNA was synthesized from 500 ng of total RNA by ReverTra ACE qPCR RT Master Mix with gDNA Remover (TOYOBO, Japan). Quantitative RT-PCR was performed according to the manufacturer’s instructions by diluting the synthesized cDNA ten folds and using a KAPA SYBR FAST qPCR Kit (KAPA Biosystems, United States) and a ViiA7 real-time PCR system (Thermo Fisher Scientific, United States). The qRT-PCR conditions were as follows: (1) initial denaturation at 95°C for 30 s; (2) 40 cycles of denaturation at 95°C for 5 s; and (3) final annealing and elongation at 60°C for 30 s. Expression levels were standardized using a VPS-like gene (Glyma.09G196600). The primer pairs used for quantitative RT-PCR are listed in Supplementary Table S2.

### Biochemical Parameters

Nitrogen content (*N_cont_*), Chlorophyll a (*Chl a*), Chlorophyll b (*Chl b*), and Chlorophyll a + b (*Chl a + b*) were quantified at the same leaves where the gas exchange was measured. For measuring *Chl a*, *Chl b*, and *Chl a + b*; four-leaf disks of 0.5 cm diameter were taken on 30 July and dissolved in 2 ml N, N-Dimethylformamide (DMF) as described by Porra (2002). The samples were then wrapped in aluminum foil and stored at 5°C for over 24 h. Spectroscopic readings of the supernatant were taken at 646.8 nm, 663.8 nm, and 750 nm wavelengths using a U-2910 Spectrophotometer (HITACHI, Japan). Nitrogen content was measured on 30 July and 11 August. The leaf area was measured using a leaf area meter LI-3100C (LI-COR, United States). They were then oven-dried for 72 h and were used for quantifying *N_cont_* using Kjeldahl digestion. We followed the method of Vickery (1946) for quantifying *N_cont_*. The oven-dried leaves were crushed, and a sample of 0.2 g was dissolved and heated in 4 ml of highly concentrated sulfuric acid until the solution turned colorless. The colorless solution was then diluted to 40 ml with distilled water. We separated 20 μl aliquots of this solution into glass tubes and mixed it with 2.48 ml of distilled water, 1 ml of Indophenol A, and 1.5 ml of Indophenol B. These were then read at 635 nm wavelength using a U-1100 Spectrophotometer (HITACHI, Japan).

### Morphological Parameters

Stomatal density (*S_Density_*), stomatal length (*S_Length_*), and stomatal width (*S_Width_*) were measured on 30 July and 11 August using the same leaves used for the gas exchange measurements. We followed Tanaka and Shiraiwa (2009) for determining stomatal parameters. After taking disks for chlorophyll content analysis, we used the Suzuki Universal Method of Printing (SUMP) for printing stomatal maps. A droplet (around 10 μl) of SUMP liquid (amyl acetate) was placed on a SUMP disk. The leaf was placed on the disk and left to air-dry for ~20 min. The disks were later observed at 100X (for *S_Density_*) and 400X (for *S_Length_* and *S_Width_*) magnifications under light microscope BH-32 (OLYMPUS, Japan) with a Multi-Interface Digital Camera FLOYD (Wraymar, Japan). ImageJ was used for counting stomata and measuring their length and width. We also measured specific leaf weight (*SLW*) as an alias to leaf thickness which is the ratio of a leaf’s dry weight to its area (g m^−2^).

### Statistical Tests and Graphs

Two-way ANOVA and Tukey’s Honest Significant Difference (Tukey HSD) analysis were conducted for all parameters using the built-in R functions of “aov” and “TukeyHSD”; and “HSD.test” function of Agricolae package (De Mendiburu, 2015). Accessions (G) and the measurement dates (D) were used as factors. Due to field homogeneity, we considered block difference as negligible. For correlations among parameters, we used the “pearsonr” function of the Scipy library (Pauli et al., 2020) in Python. We used the Pythons matplotlib.pyplot (Hunter, 2007) library for creating figures; and Scikit learn library (Pedregosa et al., 2011) for K-means clustering.

## Results

### Screening, Distribution, and Clustering of *A_sat_*

**Figure 1:**
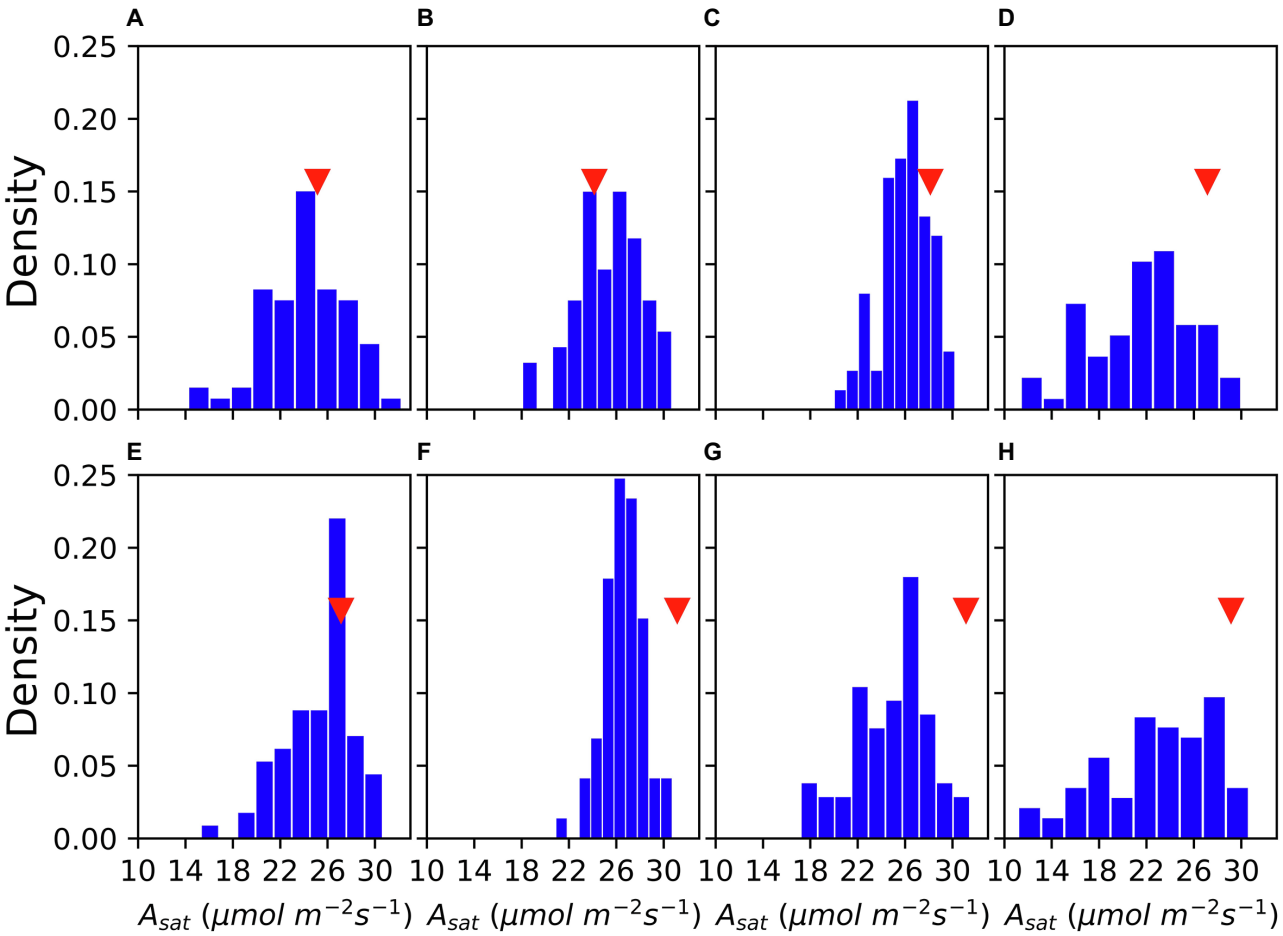
Variation of *A*_sat_ in the GmJMC (JMC) accessions and its comparison with Fukuyutaka (Fu) across measurements. 5 August, 2019 **(A)** 13 August, 2019 **(B)** 18 August, 2019 **(C)** 6 August, 2020 **(D)** 14 August, 2020 **(E)** 21 August, 2020 **(F)** 27 August, 2020 **(G)** and 31 August, 2020 **(H)**. Red triangles show the *A*_sat_ performance of Fu.

**Figure 2:**
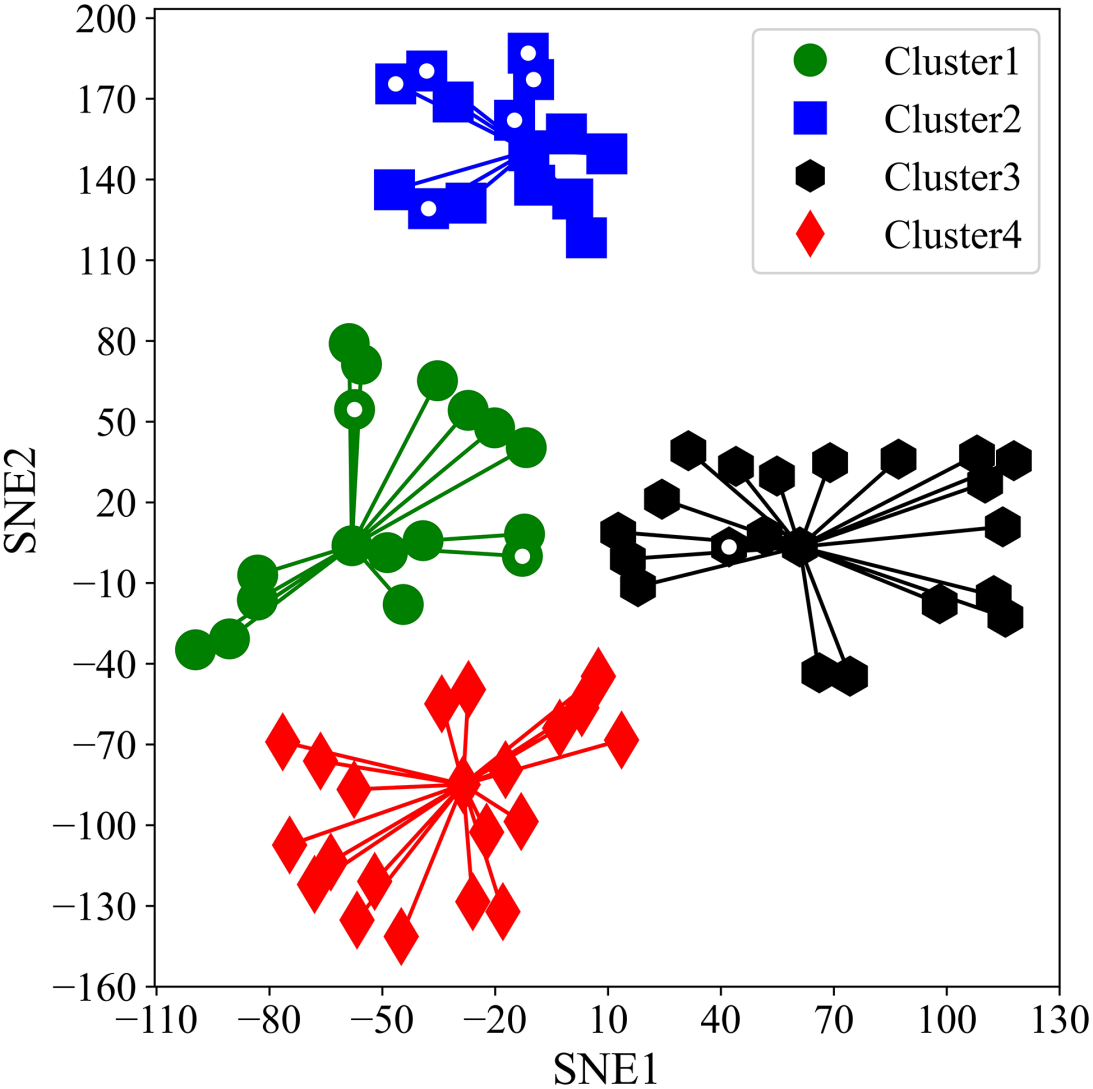
Two-dimensional scatter plot of four different K-means clusters alongside SNE1 and SNE2. White dots inside markers indicate representative accessions we used for our second experiment.

As shown in Figure 1, there were great variations in *A_sat_* among accessions and measurement dates. *A_sat_* ranged from 10 μmol m^−2^ s^−1^–33 μmol m^−2^ s^−1^. Both in 2019 and 2020, the frequency of accessions with lower *A_sat_* was higher at the beginning and the end of August, whereas the *A_sat_* measurements conducted in mid-August had a narrow range with most accessions showing greater *A_sat_*. Shapiro’s test of normality revealed that the data were normally distributed.

t-SNE compressed variations of *A_sat_* into two components. SNE1 explained only 22.9% variations whereas SNE2 explained 77.0% variations in *A_sat_* measurements. SNE1 had a negative correlation with all the *A_sat_* measurements and was particularly strong with measurements taken on 05 August 2019, and 06 August 2020. In contrast, SNE2 was positively correlated with all the *A_sat_* measurements (Table 1). t-SNE classified accessions into four clusters with massive differences between photosynthetic capacities (Figure 2). The computed Silhouette coefficient was 0.76 indicating that the clusters are spherical with only minor overlapping among them.

**Table 1:** Correlation coefficient between t-SNE components and *A_sat_* in each measurement date.

Date	SNE1	SNE2
5 August, 2019	−0.65	0.55
13 August, 2019	−0.13	0.52
18 August, 2019	0.13	0.57
6 August, 2020	−0.75	0.37
14 August, 2020	−0.34	0.57
21 August, 2020	−0.57	0.65
27 August, 2020	−0.13	0.76
31 August, 2020	−0.30	0.85

SNE1 and SNE2 show first and second t-SNE components, respectively.

**Figure 3:**
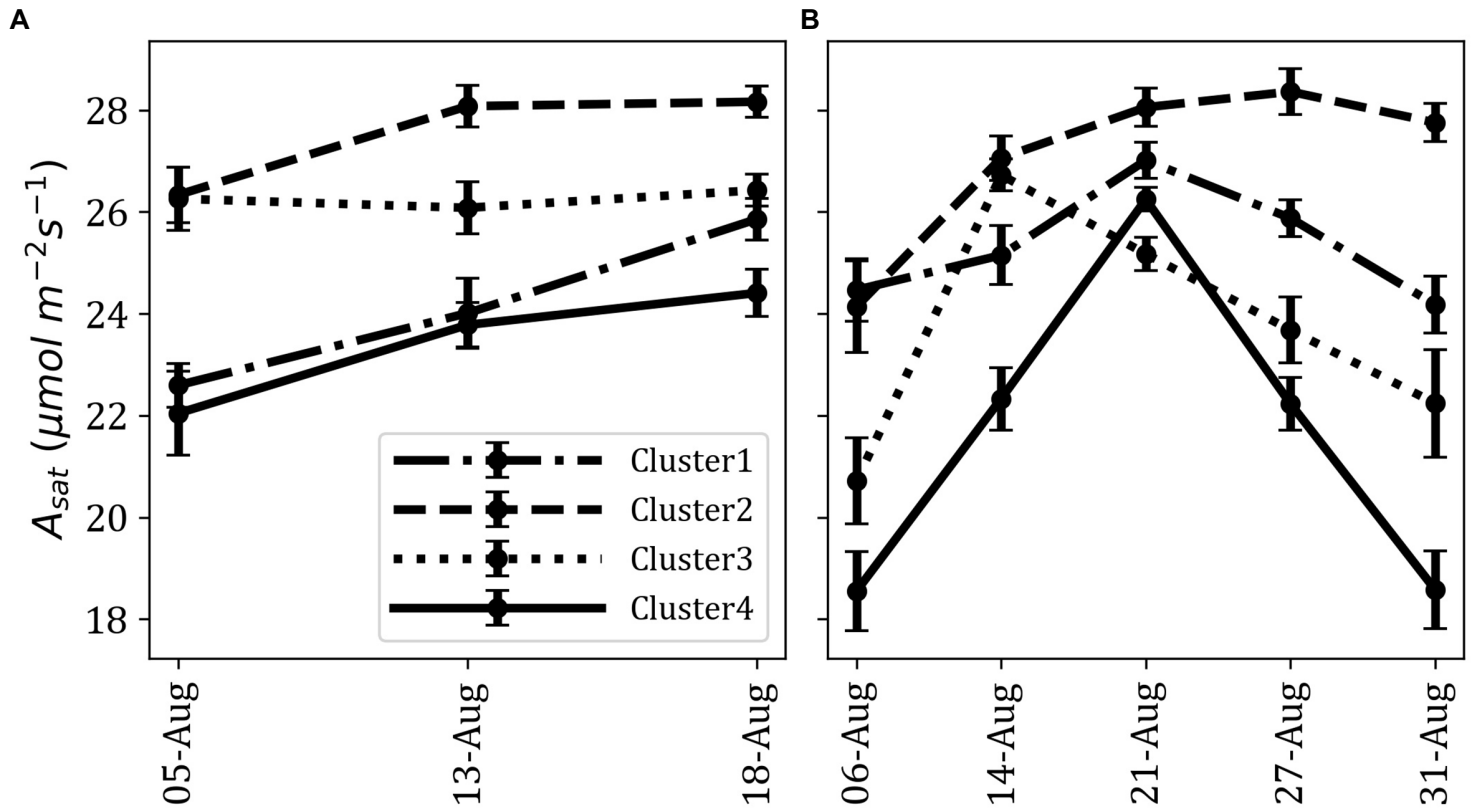
Difference of fluctuation in *A_sat_* for each cluster across measurements; average *A_sat_* in 2019 **(A)** and average *A_sat_* in 2020 **(B)**. Data point represents the mean of all members in a cluster. Error bars represent standard error.

We observed significant differences in *A_sat_* among the clusters (*p* < 0.001; Figure 3). On average, Cluster2 had the highest *A_sat_* and consisted of some of the best photosynthesizing (*A_sat_*) accessions. Some of these accessions showed photosynthetic rates comparable to or higher than the elite cultivars, En in Cluster3 and Fu in Cluster1. Cluster1 and Cluster3 had cross-year variations in their *A_sat_* values whereas Cluster4 was characterized by the lowest *A_sat_* values in 2019 and 2020.

### Genetic Characterization and RNA Expression of the Candidate Genes

**Figure 4:**
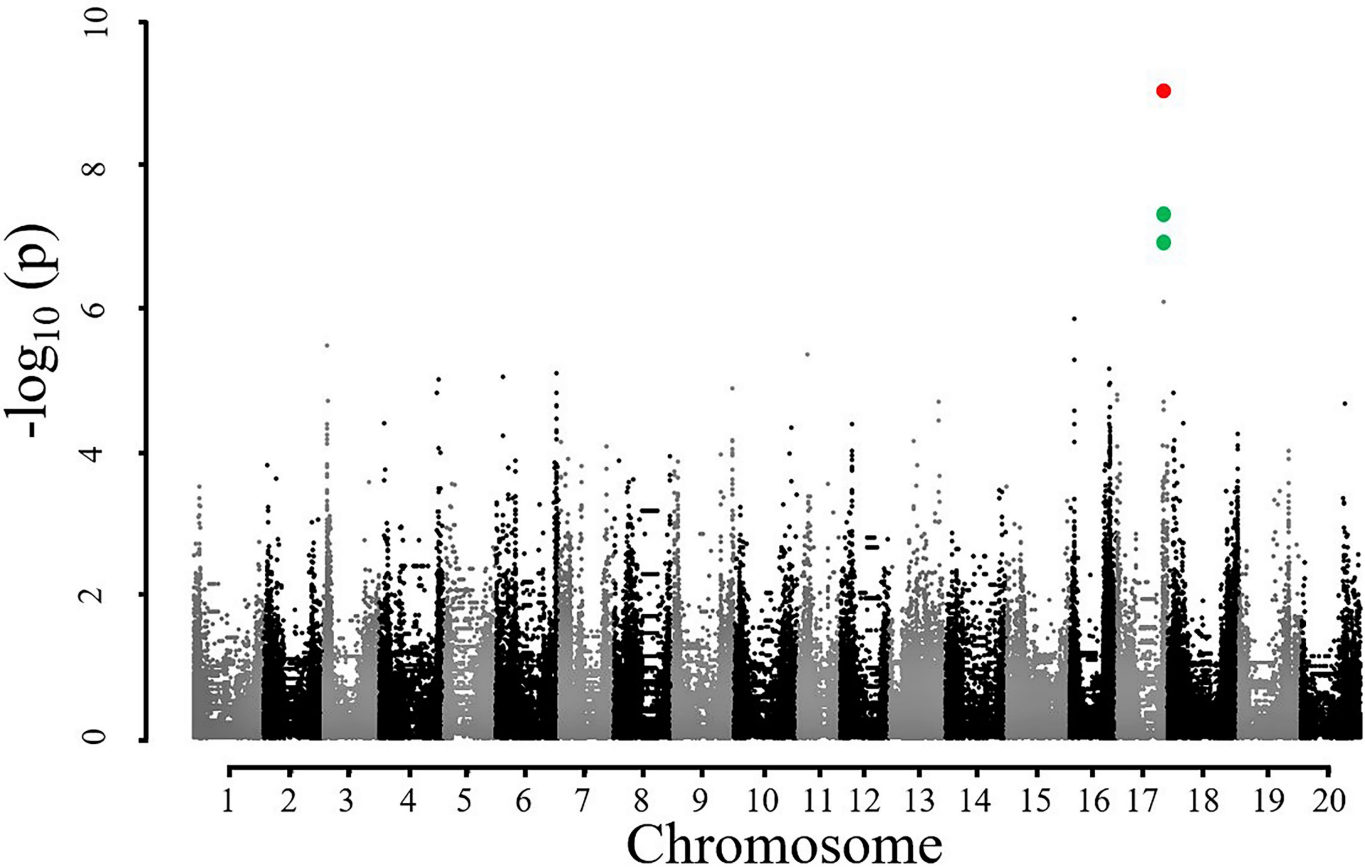
Manhattan plot for showing significant association of SNPs with SNE2. Red and green dots on chromosome 17 represent significant and suggestive associations where –log (*p*) value is less than 8 and 5, respectively.

**Figure 5:**
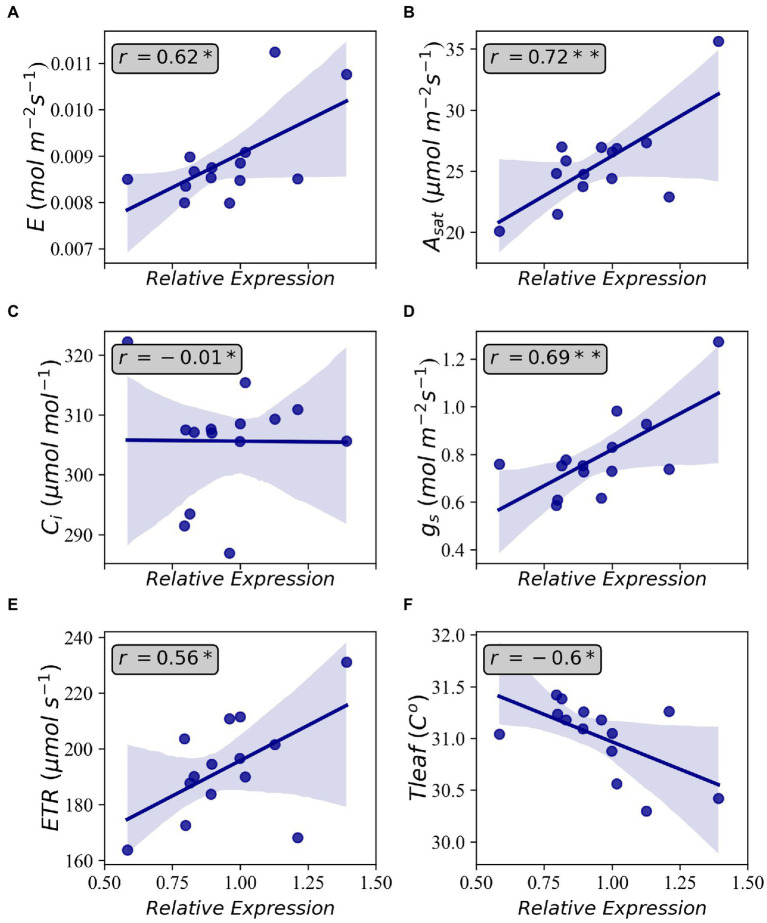
Correlations between relative gene expression of Glyma.17G226700 and Transpiration **(A)**, light-saturated photosynthesis **(B)**, intercellular CO_2_ concentration **(C)**, stomatal conductance **(D)**, electron transfer rate **(E)**, and leaf temperature **(F)**. Shaded areas indicate 95 percent confidence intervals. Annotations inside the plots indicate Pearson’s correlation coefficient. Each data point is the average of four observations. *p < 0.05 and **p < 0.01.

Based on the results of Table 1, SNE2 was interpreted to represent the genetic potential of *A_sat_*. GWAS using SNE components revealed a region on chromosome 17 that had a strong association with variations of SNE2 (Figure 4). In contrast, the GWAS result of SNE1 (Supplementary Figure S1) was not considered for further analysis due to a lower -log (*p*) value and few explainable variations for *A_sat_* (Table 1). In a 500 kbp region spanning both sides of the significant peak detected in GWAS of SNE2, 42 genes are annotated (Supplementary Table S1). Among them, 19 genes were proteins that are assumed to be involved in photosynthesis-related functions. RNA expression analysis of the three candidate genes, Glyma.17G226100, Glyma.17G226700, and Glyma.17G226900, revealed a strong correlation between the expression levels of Glyma.17G226700, and *A_sat_* (*r* = 0.72), *g_s_* (*r* = 0.69), Transpiration or E (*r* = 0.62), ETR (*r* = 0.56), and leaf temperature or Tleaf (*r* = −0.60); however, there was no correlation between the expression of this gene and *intercellular CO_2_ concentration* (*C_i_*; Figure 5). The correlation of Glyma.17G226100 and Glyma.17G226900 with *A_sat_* is shown in Supplementary Figure S2.

### Gas Exchange and Chlorophyll Fluorescence

**Figure 6:**
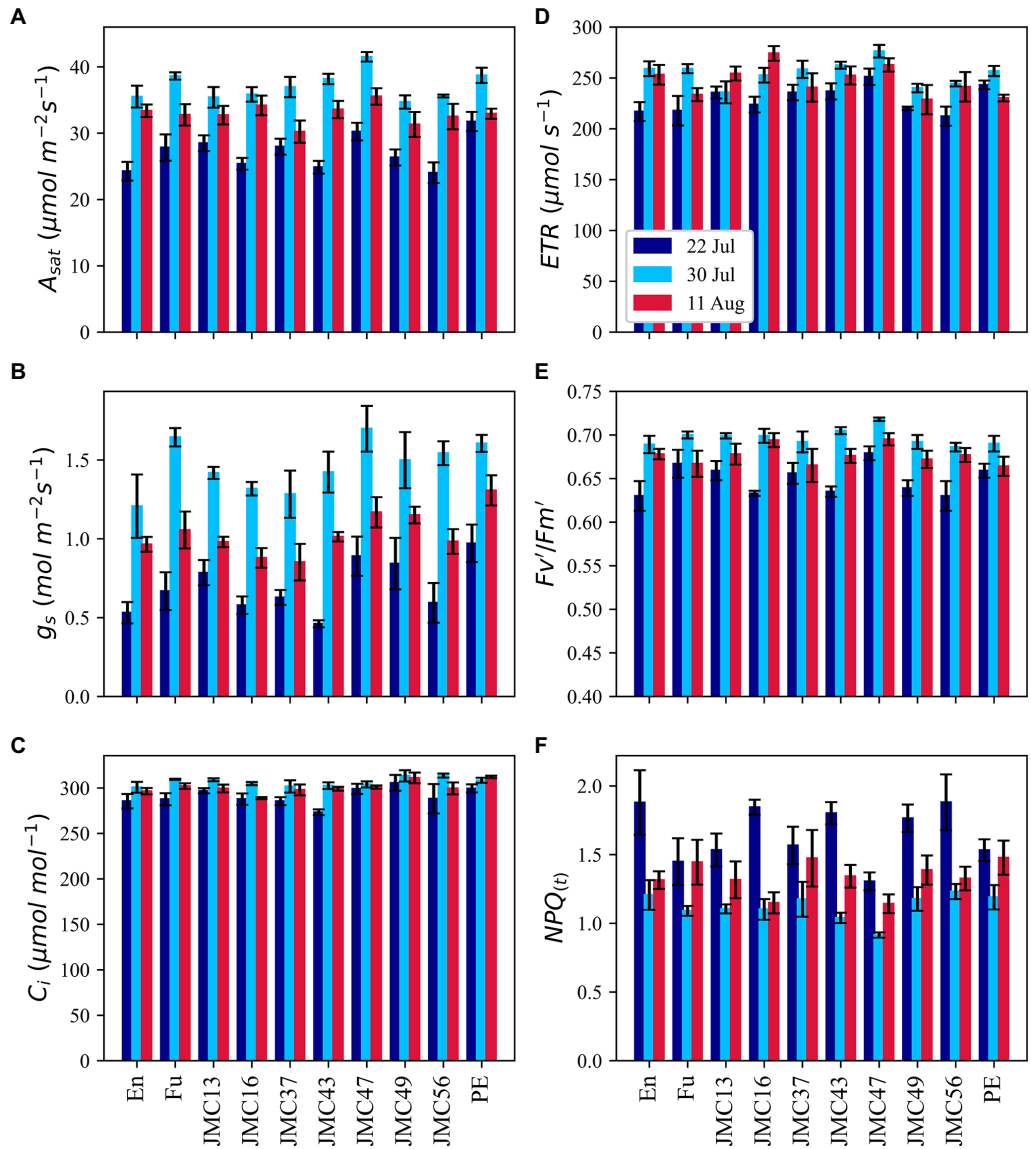
Phenotypic variations in gas exchange and chlorophyll fluorescence parameters; light-saturated photosynthetic rate **(A)**, stomatal conductance **(B)**, intercellular CO_2_ concentration **(C)**, electron transport rate **(D)**, the light utilization efficiency of *PSII* **(E)** and alias to non-photochemical quenching **(F)**. Data point is the mean of four observations. Error bars indicate standard error.

Considerable variations of *A_sat_* among 10 representative accessions and measurement dates were observed during our second experiment in 2021 (Figure 6A). *A_sat_* ranged from 24.1 m^−2^ s^−1^ in JMC56 on 22 July to 41.5 μmol m^−2^ s^−1^ in JMC47 on 30 July. The lowest *A_sat_* was observed on 22 July which then increased sharply on 30 July followed by a drop on 11 August. Among accessions, PE had the highest *A_sat_* on 22 July followed by JMC47. PE and JMC47 were significantly higher than En, JMC43, and JMC56 (*p* < 0.05). On 30 July, JMC47 showed the highest *A_sat_* and was significantly greater than En, JMC13, JMC16, JMC49, and JMC56 (*p* < 0.01). It was also the best performing accession on 11 August, but the difference was not significant among accessions. The *g_s_* measurements showed a similar pattern to *A_sat_*, but with greater magnitudes (Figure 6B). It ranged from 0.46 in JMC43 on 22 July to 1.69 mol m^−2^ s^−1^ in JMC47 on 30 July. The *g_s_* values was exceptionally low on 22 July followed by a sharp increase on 30 July and a drop on 11 August. PE and JMC47 had the highest *g_s_* values in all measurements. Seasonal change in *C_i_* was also comparable to *A_sat_* and *g_s_*. The lowest rates of *C_i_* were recorded on 22 July, followed by an increase on 30 July and a drop on 11 August (Figure 6C). Among accessions, JMC43 had the lowest *C_i_* of 273.3 μmol mol^−1^ on 22 July, whereas JMC56 had a *C_i_* of 313.6 μmol mol^−1^ on 22 July. We also calculated the apparent mesophyll activity (*A*/*C_i_*) and found that its variations were strongly correlated with variations in *A_sat_* (Supplementary Table S3). JMC47 had the highest *A/C_i_* values in all measurements whereas En and JMC56 had the lowest *A/C_i_* on 22 July.

Chlorophyll fluorescence parameters exhibited a similar pattern to gas exchange parameters. On average, *ETR* was significantly lower on 22 July than 30 July and 11 (*p* < 0.05) but the values were not significantly different between 30 July and 11 August (Figure 6D). *ETR* ranged from 212.1 μmol m^−2^ s^−1^ in JMC56 to 276.2 μmol m^−2^ s^−1^ in JMC47. *FV’/Fm′* rates were also the lowest on 22 July followed by an increase on 30 July and a drop on 11 August (Figure 6E). JMC47 exhibited the highest *Fv’*/*Fm′* among all the measurements. In contrast, *NPQ*(*t*) exhibited a reverse pattern of gas exchange and fluorescence parameters (Figure 6F). Its mean values were 1.65, 1.12, and 1.33 on 22 July, 30 July, and 11 August, respectively, and the difference was significant (*p* < 0.01). It ranged from 0.91 in JMC47 on 30 July to 1.8 in En on 22 July. JMC47 had significantly low *NPQ*(*t*) values on 22 July and 30 July among all the accessions (*p* < 0.01).

*A_sat_* was strongly correlated with *g_s_* in all the measurements; however, the strength of correlation was weaker on 11 August (Supplementary Figure S4). The positive correlation between *A_sat_* and *C_i_* was significant on 22 July (*p* < 0.01) whereas it became negatively correlated in the last two measurements. Interestingly, the positive correlation between *C_i_* and *g_s_* was constantly observed across all the measurements. *ETR*, *Fv’/Fm′*, and *PhiPS2* had strong correlations with *A_sat_*. On contrary, *NPQ*(*t*) was negatively correlated with *A_sat_* and other Chlorophyll fluorescence parameters.

### Chlorophyll and Nitrogen Content

Variations in *Chl a*, *Chl b,* and *Chl a + b* contents were observed among accessions. En, JMC49, and JMC56 are distinguished with lower *Chl a, Chl b, and Chl a + b* contents. *Chl a* was significantly (*p* < 0.05) higher in PE than in En and JMC56, and *Chl b* was significantly higher in PE than En, JMC13, and JMC56 (*p* < 0.05; Supplementary Figure S3A). JMC47 had the highest *Chl a, Chl b, and Chl a + b* contents among the JMC accessions and was comparable with that of PE. We observed consistency of *N_cont_* variation among accessions between 30 July and 11 August except for JMC16 and JMC56 (Supplementary Figure S3B). The lowest *N_cont_* of 1.25 gm^−2^ was seen in JMC37 and JMC43 whereas JMC56 had a higher *N_cont_* value of 1.92 g m^−2^ on 30 July than that of most accessions. We did not observe any significant difference in *N_cont_* values on 11 August. On average, *N_cont_* was higher on 30 July than on 11 August.

The correlation between *Chl a, Chl b, and Chl a + b* and *A_sat_* was significant (*p* < 0.01; Supplementary Figure S4). However, we did not observe a significant correlation between chlorophyll contents and chlorophyll fluorescence parameters.

### Stomatal Attributes and Specific Leaf Weight

**Figure 7:**
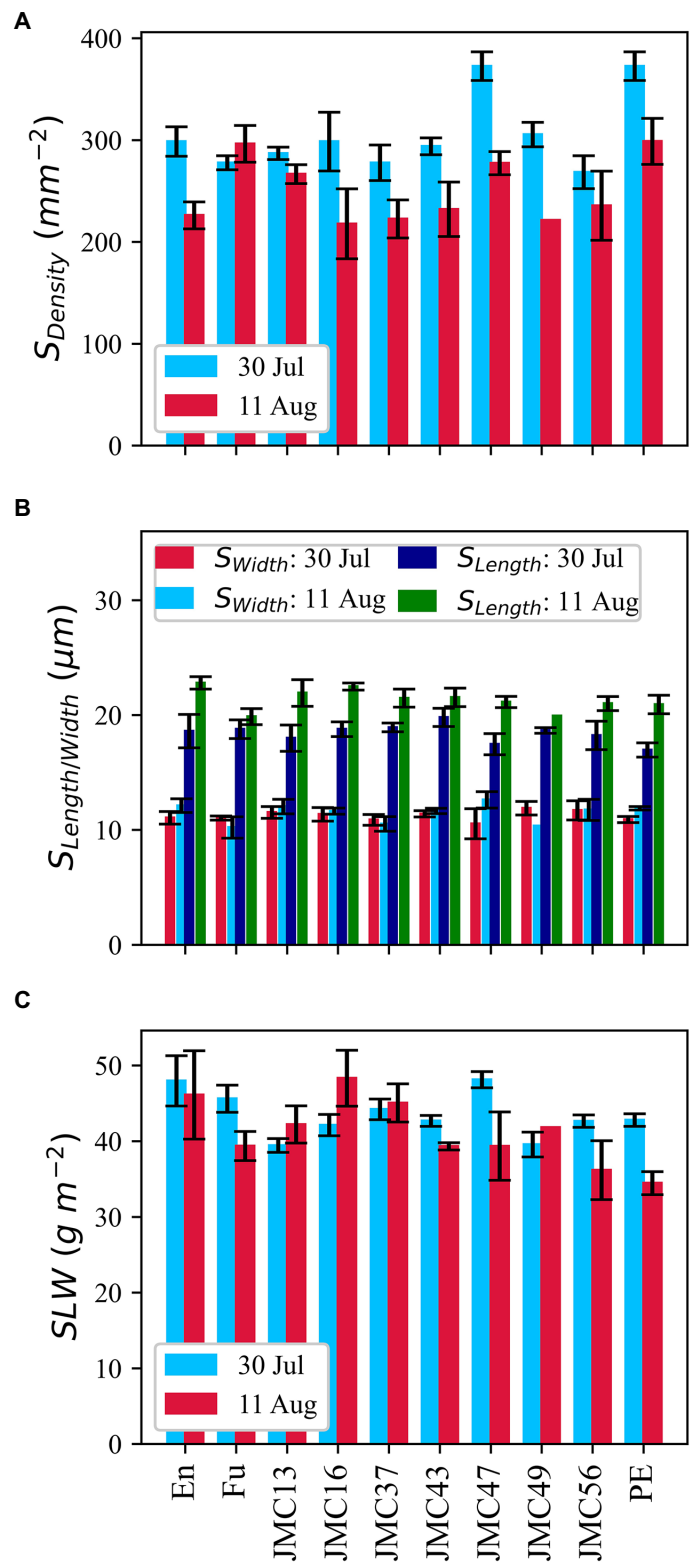
Phenotypic variations in morphological parameters of stomatal density **(A)**, the length and width of stomata **(B)**, and specific leaf weight **(C)**. Data point is the mean of four observations. Error bars indicate standard errors.

JMC47 had the highest *S_Density_* among the JMC accessions on 30 July (Figure 7A). PE and JMC47 had significantly more stomata than Fu, JMC13, JMC37, and JMC43 (*p* < 0.05). The *S_Density_* difference among accessions was not clear on 11 August. Variations of *S_Length_* and *S_Width_* (Figure 7B) were also observed among accessions and between measurements, but the difference was only significant between the measurement dates (*p* < 0.05) and not among the accessions. *S_Length_* was twofold longer than *S_Width_* for all the accessions.

En and JMC47 showed distinguishably higher specific leaf weight (*SLW*) on 30 Jul, however, there was no consistency between the values of 30 July and 11 August (Figure 7C). JMC13 and JMC49 had smaller *SLW* values than other accessions on 30 July; however, on 11 August, PE and JMC56 had the lowest *SLW* values. We observed accessions (G) to measurement date (D) interaction at *p* < 0.05, but the average *SLW* values were neither significant among accessions nor between the two measurement dates.

A positive correlation between gas exchange parameter *g_s_* and *S_Density_* was observed (Supplementary Figure S4) but it was only significant on 11 August (*p* < 0.05). A strong negative correlation existed between *S_Density_* and *S_Length_* whereas the correlation between *S_Density_* and *S_Width_* was not significant. *S_Length_* was also negatively correlated with *A_sat_*, *C_i_*, and *g_s_*. *SLW* was not correlated with *A_sat_* in pooled data, nor was it with individual measurements.

## Discussion

The culmination of conventional yield improving parameters has widened the margin between food demand and crop yield (Zhu et al., 2008, 2010), leaving the potential yield productivity to be bridged by the manipulation of photosynthetic processes in plants (Long et al., 2006; Kromdijk et al., 2016). Although exploiting natural variations of photosynthetic apparatus is considered promising for increasing crop productivity (Heckmann et al., 2017), unexplored germplasm is believed to be a major bottleneck (Kromdijk and Long, 2016) toward achieving it. We studied leaf photosynthetic capacity in JMC to (1) harness natural genotypic variations of photosynthetic capacity; (2) study the underlying genetic architecture of light-saturated photosynthesis; (3) identify physiological traits underpinning photosynthetic variations. This study is the first to assess JMC for photosynthetic studies on this scale.

### t-SNE and K-Means Clustering

As photosynthesis is a physiological process that could be affected by various environmental factors, we considered it was of importance to conduct multiple and repeated measurements and then explain total photosynthetic variations over a time course *via* dimensionality reduction. The use of MIC-100 enabled us to measure *A_sat_* repeatedly in a stunningly high number of plants which is not possible using conventional gas exchange measurement systems. The method of aggregation and interpretation of a large amount of data through large-scale phenotyping will be important to solve the bottleneck of harnessing limited genetic variations in photosynthetic research. Dimensionality reduction combined with GWAS has previously been used by Yano et al. (2019). The use of t-SNE has important implications. t-SNE is one of the best dimensionality reduction algorithms that work well for both linear and non-linear data. It is also a very robust method for visualizing high dimensional data in two- or three-dimensional panels which we considered using for our analysis. Our t-SNE analysis revealed some suitable photosynthetic characteristics of the JMC. K-means clustering classified the studied accessions into four distinct clusters with varying *A_sat_* values (Figures 2, 3). The correlation between actual data and t-SNE components indicates that SNE2 represents the genetic potential of photosynthetic capacity, whereas SNE1 seems to explain seasonal variations in *A_sat_* because of their high correlation with measurements at the beginning of August in both years (Table 1). The difference in photosynthetic performance among these clusters could be attributed to some geographical or agro-morphological basis, in addition to the unexplored limitations that lie within photosynthetic machinery. Among four clusters, Cluster2 is of particular interest as its members showed their high photosynthetic rates equivalent to or higher than the photosynthetically elite cultivars, Fu in Cluster1 and En in Cluster3 (Figure 2). Higher *A_sat_* values among the JMC accessions serve as a source of evidence for the claim that harnessing natural variations of photosynthesis is an ideal route toward removing bottlenecks in this process and improving seed yield (Heckmann et al., 2017).

### Genetic Architecture of *A_sat_*

The use of SNE2 in GWAS has two important implications. Firstly, SNE2 explains a great portion of variations in *A_sat_* values, and secondly, it had a strong positive correlation with all the measurement dates (Table 1). GWAS using SNE2 identified a genomic region at chromosome 17 (Figure 4) which had a strong correlation with SNE2. The accession numbers used in the present study was relatively small for GWAS and the detected peak should be interpreted carefully. However, the repeated measurement of *A_sat_* in 8 environments across 2 years and subsequent dimensionality reduction enabled us to detect the possible genomic region controlling the photosynthetic capacity.

One of the genes near the identified SNP is Glyma.17G226700, encoding G protein alpha subunit 1 (GPA1) which binds to abscisic acid (ABA). GPA1 mutants have been reported to have increased transpiration efficiency by minimizing *g_s_* and stomatal density (Nilson and Assmann, 2010). The loss-of-function mutation of GPA1 led to a decrease in stomatal density in the lower epidermis of *Arabidopsis* cotyledons suggesting that GPA1 positively regulates stomatal density in response to environmental and developmental cues (Zhang et al., 2008). GPA1 has also been reported to be dynamically regulating chloroplast development and response mechanism to both intracellular and intercellular signals (Zhang et al., 2009). In a previous study on soybean, Glyma.17G226700 was also detected through GWAS of photosynthetic traits concerning phosphorus efficiency (Lü et al., 2018). The positive relationship between the gene expression level of Glyma.17G226700 and *A_sat_* (Figure 5B) in this study suggests that GPA1 is an important factor in determining the natural variation of photosynthetic capacity in soybean. Nevertheless, further analysis on elucidating the functions of this gene in regulating photosynthesis is needed to utilize genetic resources in future breeding programs. Other recent studies focusing on the genetic architecture of photosynthetic capacity have also reported several loci. None of them, however, overlapped with our study. Lopez et al. (2019) identified loci related to photosynthetic capacity on chromosomes 3 and 15 in the NAM population of the US soybean. Wang et al. (2020) also reported loci affecting photosynthetic rate on chromosomes 6, 16, 18, and 19 in Chinese soybean germplasm. Yang et al. (2022) identified some loci controlling chlorophyll fluorescence on chromosomes 2, 4, 7, 8, 14, 15, 16, 18, 19, and 20 in Chinese soybean germplasm. The detection of various genomic regions between previous reports and our study suggests the complex genetic regulation and environmental interaction of soybean leaf photosynthesis.

### *A_sat_* and Physiological Mechanism Underpinning Photosynthetic Capacity

Photosynthetic capacity of the JMC accessions, in general, was highly dependent on CO_2_ diffusion, mainly due to stomatal limitations (Figure 6). Low *g_s_* on 22 July could be attributed to the early vegetative stages of the accessions. The implication of *g_s_* on increasing photosynthetic capacity is well-established (Cornish et al., 1991; Fay et al., 1995; Taylor and Long, 2017; Yamori et al., 2020). The correlation between *g_s_* and *S_Density_* (Supplementary Figures S4B,C) and the higher *g_s_* and *S_Density_* in JMC47 and PE indicates that higher *g_s_* in JMC47 and PE was due to their leaves allocating more surface to stomata. Our results are consistent with the findings of Franks et al. (2009) and Tanaka et al. (2010) also reported a strong correlation between potential stomatal conductance and *S_Density_* and argued that it could be underpinning the physiological potential for greater productivity in the U.S. soybean cultivars. In this study, JMC accessions with higher *A_sat_* had higher *g_s_* and higher *g_s_* was usually accompanied by a higher *S_Density_* (Figure 7). This indicates that Japanese soybean may show greater photosynthetic capacity if their stomatal gs is manipulated.

The best performance of JMC47 throughout vegetative and reproductive growth stages appears to be due to enhanced light utilization in addition to higher *g_s_*. This accession displayed the highest *ETR*, *Fv’*/*Fm′*, *PhiPSII*, and the lowest *NPQ*(*t*) values (Figure 6) indicating that it utilizes sunlight much more efficiently than the elite cultivar of Fu, En, and PE. The continuous superiority of *ETR* and *Fv’*/*Fm′* in JMC47 across measurement dates is consistent with findings that the application of 24-epibrassinolide increased *ETR* and the net-photosynthetic rates under water-deficient conditions and suggested that plants with higher *ETR* are more efficient in photosynthesis (Pereira et al., 2019). Sheng et al. (2008) studied the effect of *Arbuscular mycorrhiza* (AM) on photosynthesis and water status in maize and found that enhancement in photosynthetic capacity was due to enhanced *Fv’*/*Fm′*, *Fv*/*Fm*, and *PhiPSII*, and lower non-photochemical quenching (*NPQ*). Although we did not use any treatment, the natural superiority of JMC47 in showing greater *Fv’/Fm′* could be utilized as a potential resource in enhancing the light use efficiency of the photosynthetic process.

The relationship between chlorophyll content and photosynthesis has been studied intensively (Buttery and Buzzell, 1977; Garty et al., 2001; Li et al., 2006; Holly et al., 2017) and a strong positive correlation between leaf chlorophyll content and photosynthesis has been reported. A positive correlation (Supplementary Figure S4B) between chlorophyll content (*Chl a*, *Chl b*, *Chl a + b*) and *A_sat_* falls in agreement with previous findings. Higher *A_sat_* in JMC47 could also be related to its increased chlorophyll content; however, the higher chlorophyll content in PE but lower *A_sat_* compared to that of JMC47 may be due to other yet unidentified factors*. N_cont_* and *SLW* are correlated with *A_sat_* (Allison et al., 1997; Boussadia et al., 2010; Sakoda et al., 2016; Shamim et al., 2021). In our study, however, we did not observe any correlation. These results indicate that JMC47 had both stomatal and light utilization advantages over other accessions that led to its highest photosynthetic capacity.

This is the first study that reports detailed information about the photosynthetic capacity of Japanese soybean accessions together with physiological mechanism and genetic architecture underpinning photosynthesis. Here, we classified Japanese soybean germplasm into four clusters with Cluster2 comprising highly photosynthesizing accessions. GWAS revealed a locus on chromosome 17 and further genomic studies suggested the involvement of a gene, Glyma.17G226700, whose expression had a strong positive correlation with *A_sat_*. Experiment on the ten representative accessions revealed that there were stomatal and non-stomatal limitations on *A_sat_* with major limitations due to CO_2_ diffusion (lower *S_Density_*). Among all accessions, JMC47 had the highest *A_sat_* which could be attributed to its high *g_s_*, S_Density_, chlorophyll content, and chlorophyll fluorescence parameters. These results provide a piece of solid evidence to suggest that exploiting the genetic variations of plant germplasms is an ideal route to unlocking the resource use efficiency in photosynthesis.

## Data Availability Statement

The original contributions presented in the study are included in the article/Supplementary Material, further inquiries can be directed to the corresponding authors.

## Author Contributions

MS and YT designed the experiment. MS conducted the experiment, data analysis, and interpretation. GWAS was conducted by AK. HY analyzed RNA expression. The manuscript was written by MS with a contribution to the methodology of GWAS, Gene selection, and RNA expression from AK. AK and YT reviewed the manuscript and acquired funding. YT, AK, and TS coordinated and supervised the research. All authors contributed to the article and approved the submitted version.

## Funding

This study was supported by a grant from the Ministry of Agriculture, Forestry, and Fisheries of Japan (Smart-Breeding System for Innovative Agriculture, BAC1003) and Ministry of Education, Culture, Sport, Science, and Technology supported MS.

## Conflict of Interest

The authors declare that the research was conducted in the absence of any commercial or financial relationships that could be construed as a potential conflict of interest.

## Publisher’s Note

All claims expressed in this article are solely those of the authors and do not necessarily represent those of their affiliated organizations, or those of the publisher, the editors and the reviewers. Any product that may be evaluated in this article, or claim that may be made by its manufacturer, is not guaranteed or endorsed by the publisher.
