# Supplementary material for: Analysis of Physiological Variations and Genetic Architecture for Photosynthetic Capacity of Japanese Soybean Germplasm
Source: Front Plant Sci. 2022 Jun 29;13:910527. doi: 10.3389/fpls.2022.910527 (PMC9278873; doi:10.3389/fpls.2022.910527)
Supplement: Supplementary file 2 [file Presentation_1.pptx]

## Slide 1
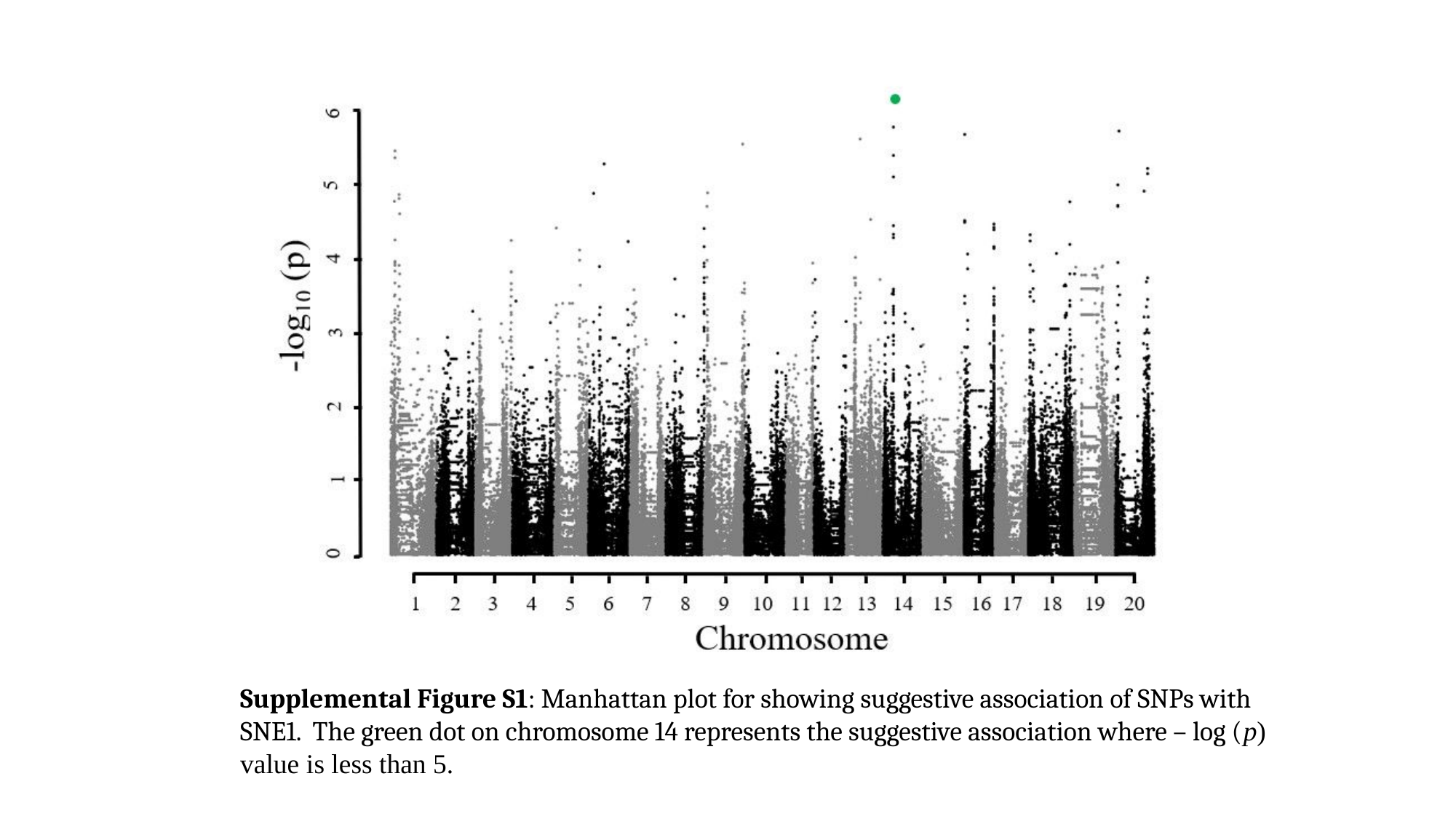

Supplemental Figure S1: Manhattan plot for showing suggestive association of SNPs with SNE1. The green dot on chromosome 14 represents the suggestive association where – log (p) value is less than 5.

## Slide 2
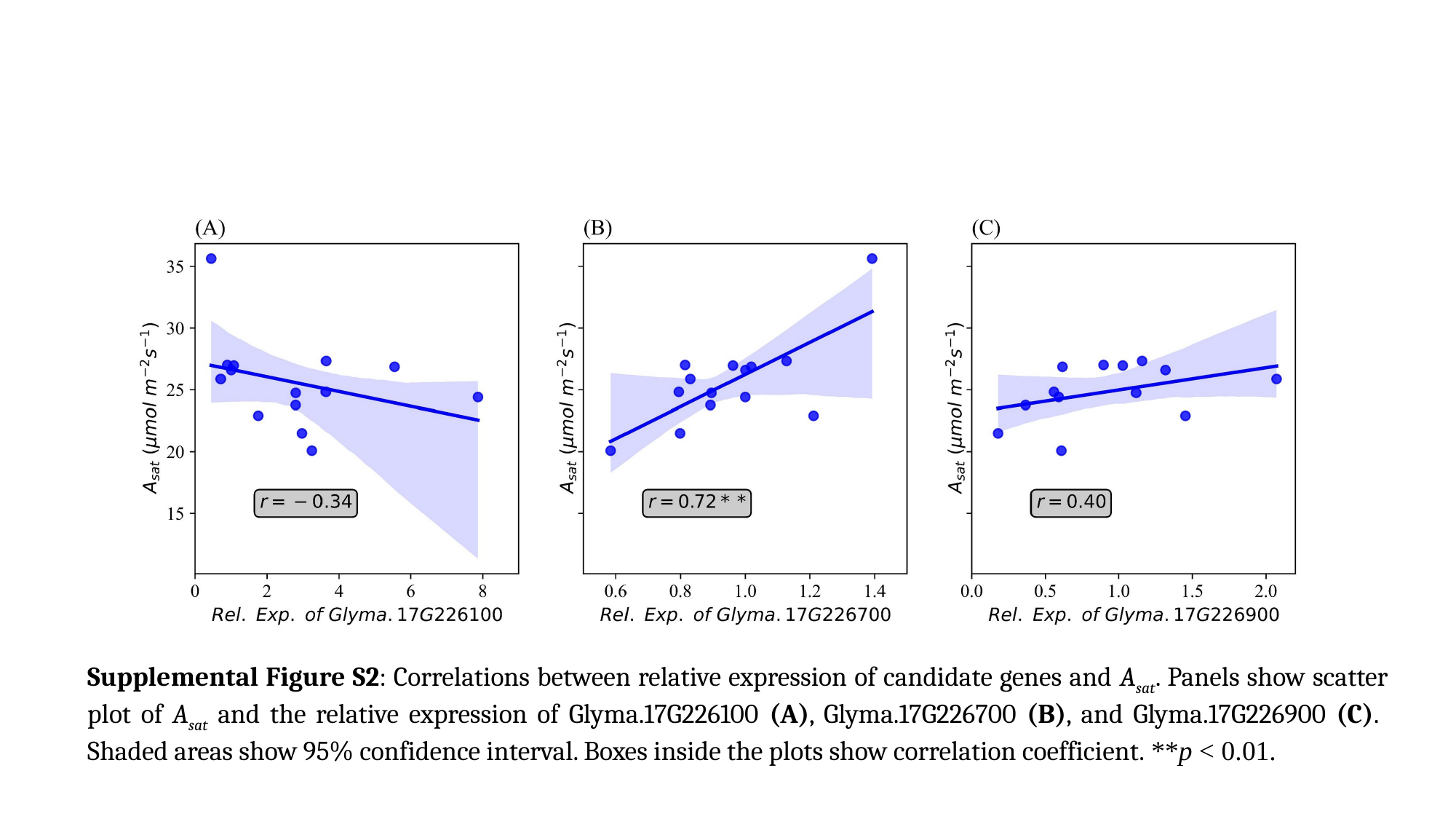

Supplemental Figure S2: Correlations between relative expression of candidate genes and Asat. Panels show scatter plot of Asat and the relative expression of Glyma.17G226100 (A), Glyma.17G226700 (B), and Glyma.17G226900 (C). Shaded areas show 95% confidence interval. Boxes inside the plots show correlation coefficient. **p < 0.01.

## Slide 3
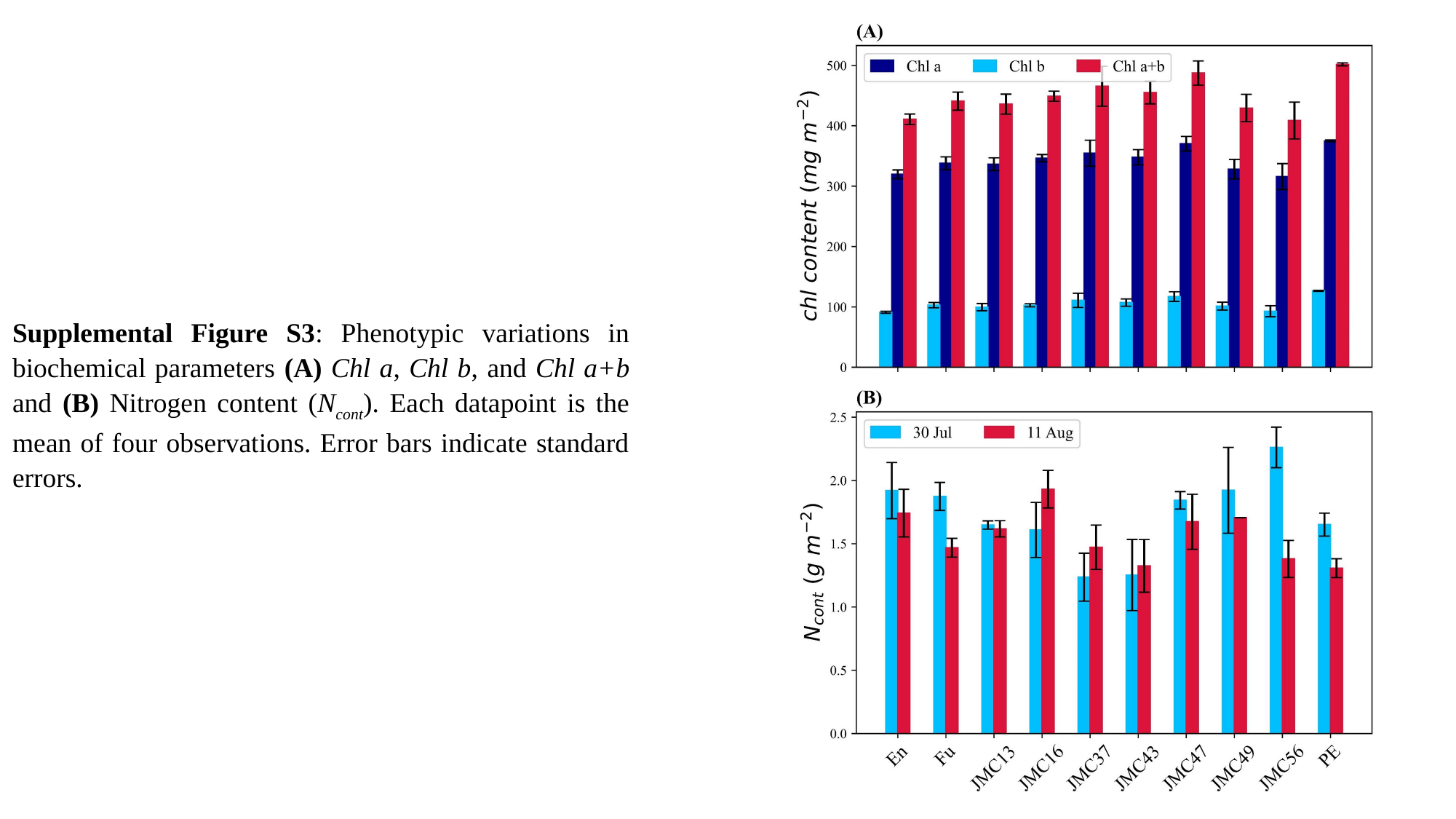

Supplemental Figure S3: Phenotypic variations in biochemical parameters (A) Chl a, Chl b, and Chl a+b and (B) Nitrogen content (Ncont). Each datapoint is the mean of four observations. Error bars indicate standard errors.

## Slide 4
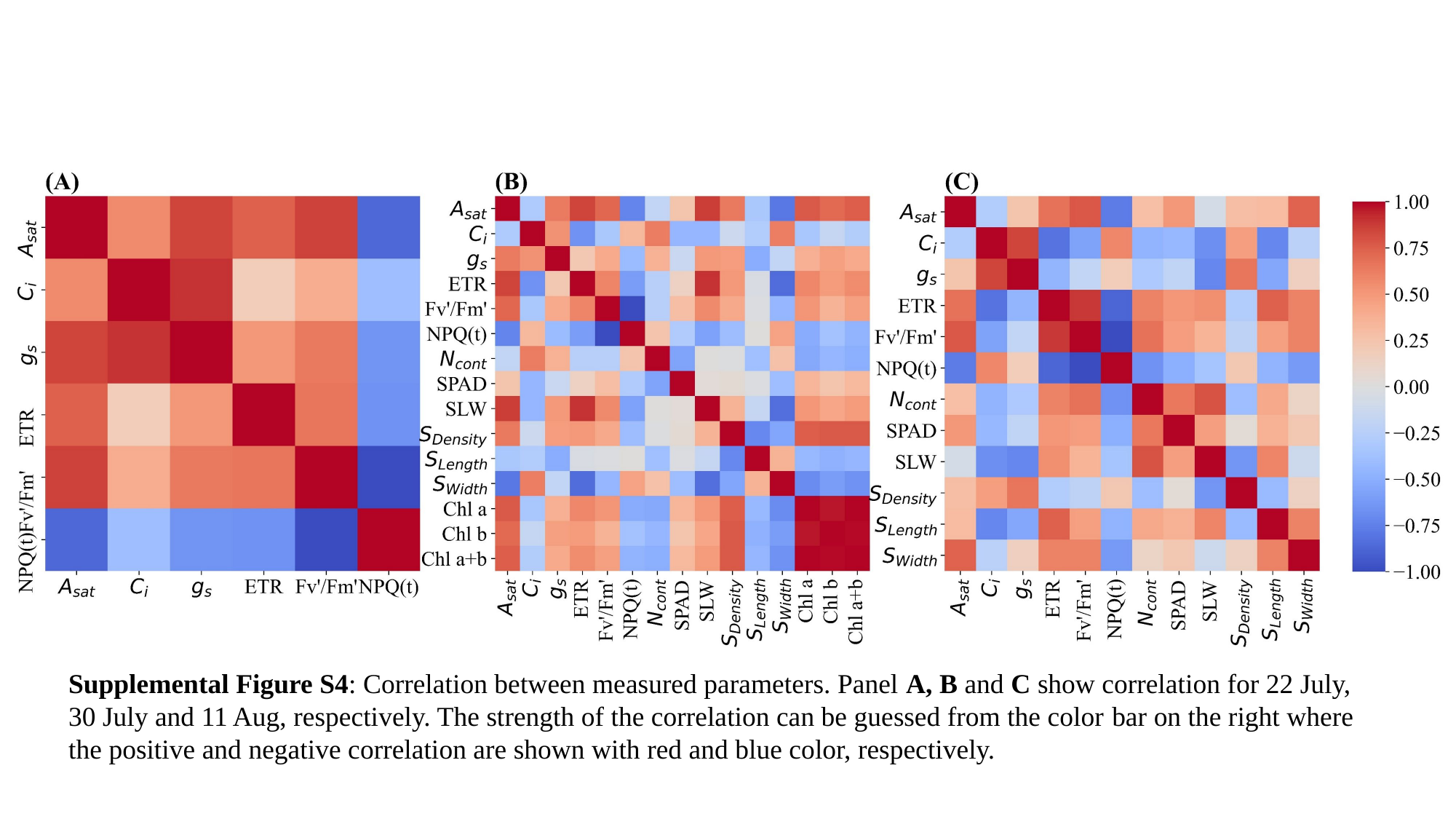

Supplemental Figure S4: Correlation between measured parameters. Panel A, B and C show correlation for 22 July, 30 July and 11 Aug, respectively. The strength of the correlation can be guessed from the color bar on the right where the positive and negative correlation are shown with red and blue color, respectively.
